# Quantification of tumor-derived cell free DNA(cfDNA) by digital PCR (DigPCR) in cerebrospinal fluid of patients with BRAF^V600^ mutated malignancies

**DOI:** 10.18632/oncotarget.13397

**Published:** 2016-11-16

**Authors:** Parisa Momtaz, Elena Pentsova, Omar Abdel-Wahab, Eli Diamond, David Hyman, Taha Merghoub, Daoqi You, Billel Gasmi, Agnes Viale, Paul B. Chapman

**Affiliations:** ^1^ Department of Medicine, Memorial Sloan Kettering Cancer Center, New York, USA; ^2^ Department of Medicine, Weill Cornell Medical College, New York, USA

**Keywords:** cell free DNA, digital PCR, BRAF mutated melanoma, erdheim-chester disease, cerebrospinal fluid

## Abstract

Tumor-derived cell free DNA (cfDNA) can be detected in plasma. We hypothesized that mutated BRAF V600 cfDNA could be quantified in the cerebrospinal fluid (CSF) of patients with central nervous system (CNS) metastases. We collected CSF from patients with BRAF V600E or K-mutated melanoma (N=8) or BRAF V600E mutated Erdheim-Chester Disease (ECD) (N=3) with suspected central nervous system (CNS) involvement on the basis of neurological symptoms (10/11), MRI imaging (8/11), or both. Tumor-derived cfDNA was quantified by digital PCR in the CSF of 6/11 patients (range from 0.15-10.56 copies/μL). Conventional cytology was negative in all patients except in the two patients with markedly elevated levels of tumor-derived cfDNA. In 2 patients with serial measurements, CSF tumor-derived cfDNA levels reflected response to treatment or progressive disease. CSF tumor-derived cfDNA has the potential to serve as a diagnostic tool that complements MRI and may be more sensitive than conventional cytology.

## INTRODUCTION

It has been known that cell free DNA (cfDNA) is released into the intravascular circulation [1] at low levels ranging from ng/ml to μg/ml [2]. In cancer patients, cfDNA in the plasma is composed of DNA from both normal and cancer cells and it has been suspected that the level of tumor-derived cfDNA can correlate with tumor burden [3]. Taking advantage of tumor-specific mutations, it has been possible to use PCR and next generation sequencing techniques to detect tumor-derived cfDNA in the plasma [4–7] and cerebrospinal fluid (CSF) [7–11] of cancer patients.

*BRAF* mutated cfDNA has been identified in the plasma of patients with both *BRAF* mutated metastatic melanoma and systemic histiocytoses including Langerhans Cell Histiocytosis and Erdheim-Chester disease (ECD), a form of non-Langerhans cell histiocytosis characterized by multi-system tissue infiltration of cells derived from monocyte/macrophage lineage [4, 12, 13]. We hypothesized that, using digital PCR (DigPCR) technology to detect *BRAF* mutations, tumor-derived cfDNA could be quantified in the CSF in these patients with CNS involvement and that the level of tumor-derived cfDNA would correlate with disease burden.

We used DigPCR to quantify tumor-derived cfDNA in the CSF from patients with melanoma or ECD. All patients had a *BRAF* V600E or K somatic tumor mutation and all underwent lumbar puncture (LP) because of suspected central nervous system (CNS) involvement on the basis of neurological symptoms, MRI imaging, or both.

## RESULTS AND DISCUSSION

From November 2013 until April 2015, CSF samples from 11 patients were collected. Median age was 57 (range 40-75); 5 were men and 6 were women. Eight patients had metastatic melanoma (7 patients with mutated BRAF^V600E^ and 1 patient with BRAF^V600K^) and 3 patients had a diagnosis of BRAF^V600E^ mutated ECD (Table 1). In all 11 patients, CSF was analyzed by conventional cytology. All patients had at least one CSF sample collected for cfDNA analysis at the time of CSF cytology collection. Two patients had two samples analyzed at different time points: before treatment and during treatment. MRI of the brain was performed in all patients. Plasma or urine samples collected within two months prior to LPs were available for analysis of tumor derived cfDNA as a measure of systemic disease in 9/11 patients.

**Table 1:** Patient demographics

Pt #	Disease	Gender	Age	Sites of Involvement	BRAF Mutation
**1**	**Mel**	**M**	**45**	**Brain, lepto, LN**	**V600E**
**2**	**Mel**	**F**	**40**	**Brain, lung, liver, soft tissue, LN**	**V600E**
**3**	**Mel**	**F**	**57**	**Brain, bone, LN, soft tissue**	**V600E**
**4**	**Mel**	**M**	**56**	**Brain, LN**	**V600E**
**5**	**Mel**	**M**	**53**	**Brain, lung, liver, LN, soft tissue**	**V600K**
**6**	**Mel**	**M**	**68**	**Lepto, lung, liver, soft tissue**	**V600E**
**7**	**Mel**	**F**	**56**	**Brain, lepto, bone, lung, LN, peritoneum**	**V600E**
**8**	**Mel**	**F**	**68**	**Liver, soft tissue, LN**	**V600E**
**9**	**ECD**	**F**	**75**	**Dura, retroperitoneum, skin, bone, lung**	**V600E**
**10**	**ECD**	**M**	**75**	**Bone, retroperitoneum, peri-aorta**	**V600E**
**11**	**ECD**	**F**	**66**	**Brain, dura, bone, peri-aorta**	**V600E**

Mel: Melanoma, ECD: Erdheim-Chester Disease, lepto: leptomeninges, LN: lymph nodes.

Ten of the 11 patients had neurologic symptoms suggestive of CNS metastases (Table 2). MRI imaging was notable for metastatic brain parenchymal disease in 7/11 and leptomeningeal disease in 3/11. Two of these patients had both brain parenchymal and leptomeningeal involvement. Conventional CSF cytology was positive for malignant cells in only 2/11 (18%) patients; both of these patients had leptomeningeal disease by MRI imaging. Conventional CSF cytology was negative in the other patients, including one patient (Patient #1) with radiographically-evident leptomeningeal disease.

**Table 2:** Pre-treatment diagnostics

Pt #	Disease	MRI: brain parenchyma	MRI: leptomeninges	Neurologic Symptoms	CSF Cytology	CSF mutant cfDNA (copies/μL)	Plasma/urine mutant cfDNA (copies/μL)
**1**	**Mel**	**+**	**+**	**-**	**-**	**0.512**	**P: 21**
**2**	**Mel**	**+**	**-**	**+**	**-**	**0**	**P: 0.8**
**3**	**Mel**	**+**	**-**	**+**	**-**	**0.219**	**P: 3.86**
**4**	**Mel**	**+**	**-**	**+**	**-**	**0**	**P: 0**
**5**	**Mel**	**+**	**-**	**+**	**-**	**0.15**	**P: 2.35**
**6**	**Mel**	**-**	**+**	**+**	**+**	**10.56**	**No samples**
**7**	**Mel**	**+**	**+**	**+**	**+**	**8.03**	**No samples**
**8**	**Mel**	**-**	**-**	**+**	**-**	**0**	**P: 174**
**9**	**ECD**	**-**	**-**	**+**	**-**	**0**	**U: 0.12**
**10**	**ECD**	**-**	**-**	**+**	**-**	**0.329**	**P: 0.76**
**11**	**ECD**	**+**	**-**	**+**	**-**	**0**	**P: .059**

Mel: Melanoma, ECD: Erdheim-Chester Disease, P: Plasma, U: Urine.

Tumor-derived cfDNA was detected and quantified in the CSF of 6/11 (55%) patients (5 patients with BRAF^V600E^ mutation and 1 patient with a BRAF^V600K^ mutation) including all 3 patients with radiographically evident leptomeningeal disease. Interestingly, tumor-derived cfDNA was detected in the CSF of 2 patients who had brain parenchymal metastases without radiographic evidence of leptomeningeal disease (patient #3, #5) and in one ECD patient who had neurologic symptoms ultimately thought not to be due to ECD (patient #10, discussed below).

In 5/11 patients (3 melanoma patients #2, 4, 8 and 2 ECD patients #9, 11), tumor derived cfDNA was undetectable in the CSF. Three of these 5 patients had brain parenchymal metastases, 1 did not have radiographic evidence of CNS metastases, and 1 ECD patient had dural disease. None had radiographically evident leptomeningeal disease and conventional CSF cytology was negative in all 5 patients. Tumor-derived cfDNA was quantifiable in plasma or urine from 4 of the patients (Patients #2, 8, 9, 11) indicating that non-CNS tumor burden was sufficient to detect tumor-derived cfDNA in the periphery. Plasma cfDNA was not detectable in patient #4 (data previously published) [12, 13].

These data show that tumor-derived cfDNA was quantifiable in 3/3 patients with radiographic evidence of leptomeningeal metastases but in only 2/5 patients who had radiographic evidence of only brain parenchymal metastases. The highest level of tumor-derived cfDNA in CSF was seen in patients with leptomeningeal metastases. Conventional CSF cytology was less sensitive.

Although the specificity of CSF cytology is >95%, the sensitivity of CSF cytology is known to be low (<50%) [14]. Possible causes of false negatives include minimal tumor cell exfoliation into the CSF, blockage in CSF flow, and the uneven distribution of malignant cells in CSF [15, 16]. Investigators have applied other, non-quantitative methods to detect malignant cells in the CSF such as multi-marker reverse transcriptase-PCR assay [17]. However these non-quantitative methods have relatively low sensitivity and do not allow for easy comparison of tumor burden across serial samples.

Three patients had no radiographic evidence of CNS involvement; cfDNA was undetectable in the CSF of two of these patients. In patient #10 with ECD and neurological symptoms, we detected low levels of tumor-derived cfDNA in the CSF even though the neurological symptoms were ultimately attributed to nutritional deficiency (see below). Leptomeningeal disease is not known to occur in ECD. We suspect that in this patient, cfDNA from the plasma may have “leaked” into the CSF. Almost all of the protein in CSF (such as albumin) is derived from serum. It is known that in the setting of infection, inflammation, or malignancy, the blood-brain-barrier and the blood-CSF barrier can be compromised [18–20]. Further studies will be needed to determine if this is a common source of false positivity in the CSF or if it is a true positive and this “leakage” is an early sign of CNS metastasis.

**Figure 1:**
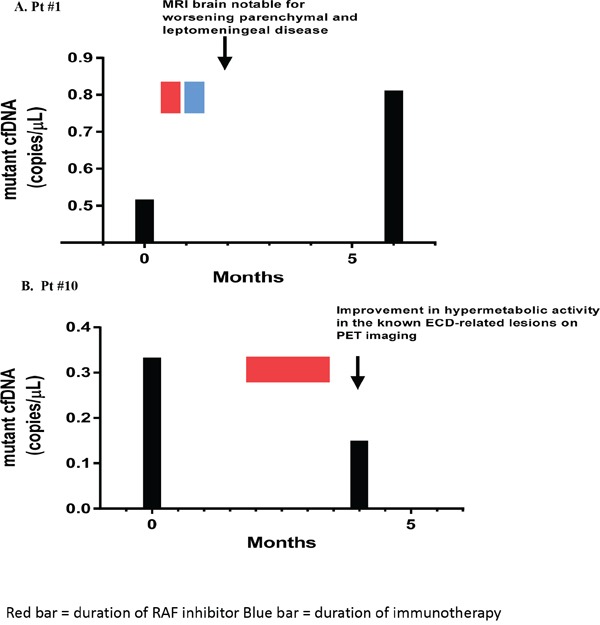
Effect of treatment on tumor-derived cfDNA in CSF **Figure 1A**. Patient #1 with melanoma. The level of mutant CSF cfDNA increased, consistent with worsening parenchymal leptomeningeal disease noted on MRI imaging. **Figure 1B**. Patient #10 with ECD. The level of tumor-derived CSF cfDNA decreased after initiation of vemurafenib, consistent with improvement in hypermetabolic activity on the known ECD-related bone lesions on PET imaging.

In two patients (1 melanoma, 1 ECD), we measured tumor-derived cfDNA in the CSF at two time points: before and during treatment (Figure 1). Patient #1 had MRI imaging notable for both brain parenchymal and leptomeningeal disease. Although the pre-treatment CSF cytology was negative for malignant cells, pre-treatment *BRAF* mutated cfDNA CSF level was measured at 0.512 copies/μL. The patient underwent treatment with dabrafenib but this had to be discontinued due toxicities. The patient then completed a course of immunotherapy with ipilimumab but developed worsening neurologic symptoms. A repeat brain MRI was notable for worsening parenchymal and leptomeningeal disease. This patient was not available for follow-up until 4 months later when a repeat lumbar puncture was performed and showed malignant cells on cytologic examination. *BRAF* mutated cfDNA in the CSF collected at that time had increased to 0.806 copes/μL. Patient #10 with ECD presented with gait imbalance and underwent CNS imaging and lumbar puncture as part of initial assessment. These were unrevealing and ultimately symptoms were found to be due to peripheral neuropathy which improved with nutritional (vitamin B12 and copper) repletion. Interestingly, CSF tumor-derived BRAF mutant cfDNA was detected pre-treatment (0.329 copies/μL). The patient was then treated with vemurafenib as part of a clinical trial. PET scan imaging showed overall improvement in hypermetabolic activity in the known ECD-related osseous lesions. During this time, the patient's disease related symptoms of bone pain and night sweats markedly improved. A repeat lumbar puncture one month into the course of treatment showed a decrease in mutant cfDNA level to 0.146 copies/μL. Both of these cases indicate that the levels of tumor-derived cfDNA in the CSF reflected response (or lack of response) to therapy.

Tumor-derived cfDNA has previously been detected in the CSF of patients with HER-2/neu positive breast cancer, glioblastoma characterized by EGFR amplification, and KRAS mutated lung adenocarcinoma using older non-quantitative PCR methods [8, 9, 11]. More recently, tumor-derived cfDNA has been detected in the CSF of patients with primary CNS malignancy and CNS metastases from solid tumors including melanoma, lung and breast cancer using next generating sequencing and the digital PCR (DigPCR) platform [7, 10].

We demonstrated that tumor-derived cfDNA can be quantified in CSF using DigPCR and that CSF cfDNA levels can reflect tumor burden and response to therapy. Although we have tested only a small number of patients, it appears that the presence of tumor-derived cfDNA in CSF is a more sensitive indicator of CNS involvement than conventional cytology. In comparison to MRI, we have observed cases with parenchymal metastases with no detectable tumor-derived cfDNA in the CSF. These cases may indicate that parenchymal metastases often do not shed DNA into the CSF. We also had a patient who was an apparent false positive; detectable tumor-derived cfDNA in the CSF with no other evidence of CNS disease. We speculate that in this patient, we were detecting cfDNA that had leaked into the CSF from the plasma. Further studies will be needed to determine if this is a common source of false positivity. If it is, then it may be possible to distinguish true positivity from false positivity by the relative level of tumor-derived cfDNA in the CSF.

## MATERIALS AND METHODS

Patients with ECD and metastatic melanoma suspected to have leptomeningeal metastasis were referred to the neurology service at Memorial Sloan Kettering Cancer Center (MSKCC) for a diagnostic lumbar puncture as part of standard of care practice. Patients signed written consent to a MSKCC biospecimen research protocol. It is standard of practice at our institution to run a standard sequenom panel that includes both BRAF^V600E^ and BRAF^V600K^ mutation analysis on metastatic tumors in patients with metastatic melanoma. BRAF^V600^ mutation information was obtained from a tissue diagnosis. Similarly for patients with ECD, mutation analysis was performed on tissue. At the time of diagnostic lumbar puncture, CSF was transferred to an EDTA vacutainer. The tube was centrifuged and processed within 2 hours of collection. CSF was transferred to sterile Eppendorf tubes in 1cc aliquots and centrifuged at 16,000 x g for 10 minutes. The supernatant was transferred into cryovials in 1cc aliquots and stored at -20C. Stored samples were then delivered for DigPCR processing 1-10 months after storage.

**Figure 2:**
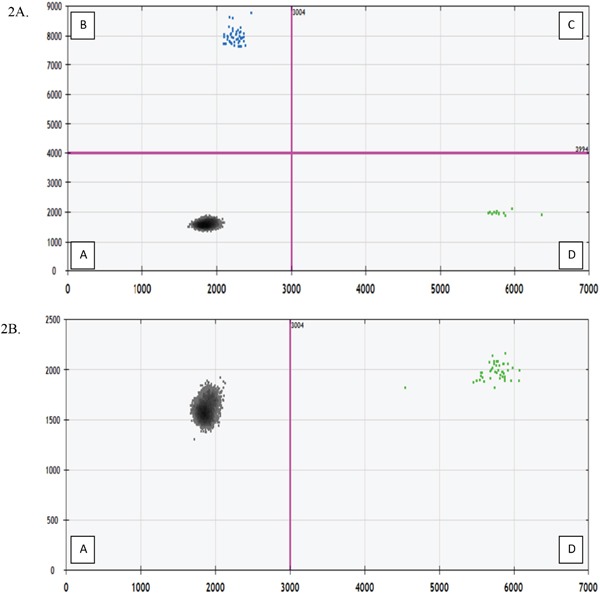
Fluorescent Amplitude Plot (X-axis: HEX flourescent wild type droplet; Y-axis: FAM fluorescent mutation droplet) Quadrant A contains the droplets with no cfDNA alleles corresponding to wild type or BRAF probe. Quadrant B contains droplets with BRAF mutated cfDNA. Quadrant C contains droplets with both wild type and mutant alleles. Quadrant D contains droplets with BRAF wild type cfDNA. **Figure 2A**. Patient #6 with detectable mutated BRAF cfDNA in CSF. **Figure 2B**. Patient #4 with undetectable mutated BRAF cfDNA in CSF.

**Figure 3:**
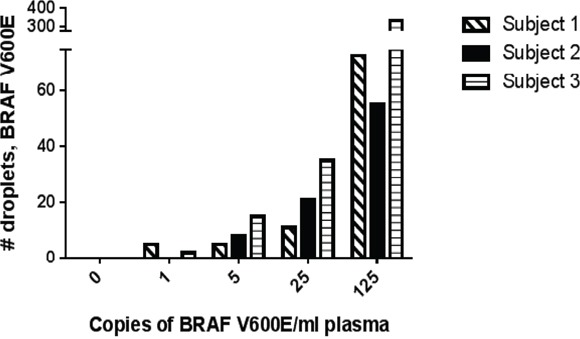
Spiking Experiment on 3 healthy volunteers to determine sensitivity of DigPCR assay 5 tubes of blood were obtained and drawn into Cell-Free DNA BCT tubes (Streck, Inc.). Appropriate serial dilutions of BRAF V600E DNA fragments were made and each tube was spiked with 1, 5, 25, or 125 copies/ml blood of BRAF V600E DNA. One tube was left un-spiked to serve as the negative control. In 2 of 3 samples, we could detect 1 molecule/ml of blood. We detected 5 molecules of BRAF V600E DNA fragments per mL of blood in all 3 samples.

Stored CSF samples were delivered on dry ice to the MSKCC Integrated Genomics Operation Core Lab. cfDNA was isolated from the samples using the Qiagen QiAamp Circulating Nucleic Acid Kit according to the manufacturer's instructions and the concentration was assessed using BioAnalyzer (100-300nt). The DNA was evaluated for BRAF V600E (1799T>A) mutation and BRAF V600K (1798_1799GT>AG) mutations using a droplet DigPCR system (BioRad QX200 Hercules, CA). Negative controls used water instead of cfDNA; the positive control used genomic DNA from a BRAF mutated cell line. The experiments were performed using the following protocol: 1 cycle at 95°C for 10 minutes, 40 cycles at 94°C for 30 seconds and 55°C for 1 minute, 1 cycle at 98°C for 10 minutes, then 1 cycle at 4°C infinite, all at a ramp rate of 2°C/second. Bio-Rad's T100 thermal cycler was used for the PCR step. The assay kits for BRAF V600E and BRAF wild-type (dHsaCP2000027, dHsaCP2000028) and BRAF V600K and wild-type (dHsaCP2000035, dHsaCP2000036) are commercially available and were purchased from Biorad and used following the manufacturer's directions. When available, 5ng of DNA was assessed in a 20μl PCR reaction, partitioned into approximately 20,000 droplets. A total of two replicates were used per sample. Droplets were quantified using the BioRad Quantasoft Software (version 7.0). For each sample, the droplet reader will read out the number of positive droplets for each florescent assay (FAM for mutant allele and HEX for wild type allele) and the software applies the Poisson distribution on the droplet count (Figure 2). The reaction volume is divided by 20μl to calculate the number of mutant droplets detected in 1μl of the reaction. The concentration is reported as copies/μl. For ECD patients, results of plasma and urine cfDNA were analyzed as previously described [12, 13]. The sensitivity of our droplet PCR assay was determined by spiking blood specimens from 3 healthy volunteers. In 2 of 3 samples, we could detect 1 molecule/ml of blood. We detected 5 molecules of BRAF V600E DNA fragments per mL of blood in all 3 samples (Figure 3).

## Abbreviations

cfDNA: cell free DNA
DigPCR: digital PCR
ECD: erdheim-chester disease
CSF: cerebrospinal fluid
CNS: central nervous system
